# The roles of long noncoding RNAs in the regulation of OCT4 expression

**DOI:** 10.1186/s13287-022-03059-9

**Published:** 2022-07-30

**Authors:** Rui-Ting Zhou, Yi-Ran Ni, Fan-Jun Zeng

**Affiliations:** 1 The First College of Clinical Medical Science, China Three Gorges University, Yichang, 443003 Hubei China; 2 Yichang Central People’s Hospital, Yichang, 443003 Hubei China; 3 Medical College, China Three Gorges University, Yichang, 443002, Hubei China; 4 Hubei Key Laboratory of Tumor Microenvironment and Immunotherapy, China Three Gorges University, Yichang, 443002 Hubei China

**Keywords:** LncRNA, OCT4, Cancer stem cell, Gene expression regulation

## Abstract

OCT4 is a major transcription factor that maintains the pluripotency of stem cells, including embryonic stem cells, induced pluripotent stem cells and cancer stem cells. An increasing number of long noncoding RNAs have been reported to participate in the regulation of OCT4 expression through various mechanisms, including binding with the OCT4 gene promoter to regulate local methylation; promoting chromosomal spatial folding to form an inner ring, thereby aggregating OCT4 cis-acting elements scattered in discontinuous sites of the chromosome; competitively binding microRNAs with OCT4 to upregulate OCT4 expression at the posttranscriptional level; and sharing a promoter with OCT4. Moreover, the transcription of some long noncoding RNAs is regulated by OCT4, and certain long noncoding RNAs form feedback regulatory loops with OCT4. In this review, we summarized the research progress of the long noncoding RNAs involved in the regulation of OCT4 expression.

## Background

Stem cells (SCs) refer to cells that have both the capacity of self-renewal and the capacity to further generate one or more differentiated cell types. There are two major types of SCs: adult stem cells, including haematopoietic stem cells (HSCs), and embryonic stem cells (ESCs), which are derived from the inner cell mass of the developing blastocyst [1]. One major difference between adult stem cells and ESCs is that a single cell of the latter can give rise to all cell lines of both developing and adult organisms, a property known as pluripotency. The pluripotent state of ESCs is precisely controlled by a group of transcriptional regulators [2]. Octamer-binding transcription factor 4 (OCT4), encoded by POU class 5 homeobox 1 (*POU5F*1) on 6p21.33, was first reported to be a key transcription factor (TF) in maintaining pluripotency of ESCs by Nichols et al. [3] as early as 1998. Later, Yamanaka et al. [4] reprogrammed fibroblasts into induced pluripotent stem cells (iPSCs) by OCT4 and three other transcription factors (SOX2, KLF4 and C-MYC). The clinical usage of ESCs is controversial and ethical and safety issues may arise, while adult stem cells grow slowly and are obtained invasively [5]. Therefore, Yamanaka's outstanding contribution of iPSCs has paved the way for stem cell therapies by addressing these constraints associated with ESCs and adult stem cells, and he won the 2012 Nobel Prize in Physiology or Medicine for this work. Reprogramming iPSCs and further differentiating iPSCs to a specialized stage by different combinations of stimulators is a popular strategy in stem cell therapy-related research [6]. Additionally, the reprogramming schemes are improving. Recently, Guan et al. successfully restored the expression of OCT4 in fibroblasts and reprogrammed them into iPSCs with a group of small molecules instead of TFs [7].

The hypothesis of cancer stem cells (CSCs) began in the mid-1990s, when a rare cell type that was capable of growing into a new neoplasm after injection into mice was isolated from the blood of patients with leukaemia. The concept of CSCs emphasizes that these cells are capable of self-renewal and thereby play a fundamental role in cancer initiation and recurrence [8]. As in maintaining pluripotency of ESCs or iPSCs, OCT4 is also a key TF that maintains the “stemness” of CSCs and is also an important marker for identifying these cells [8, 9]. In a variety of human malignancies, such as breast cancer [10], ovarian cancer [11], colon cancer [11], melanoma [11], kidney cancer [11], bladder cancer [12], and lung cancer [13], the high expression of OCT4 indicates that the tumour has a stronger ability of proliferation, invasion and metastasis and is also closely related to drug resistance and recurrence of radiotherapy and chemotherapy. Therefore, OCT4 is a potential target for cancer therapy, and the regulation of its expression is a hot topic in the research fields of development, stem cell therapy and oncology.

Long noncoding RNAs (lncRNAs), ranging in length from 200 nucleotides to 100,000 nucleotides, lack the ability to encode proteins and hence the name. It is speculated that there are 20,000 human lncRNAs, of which more than 200 have been confirmed to have clear biological functions, such as regulation of the cell cycle, cell proliferation and differentiation, nuclear cytoplasmic transport and gene transcription and translation [14]. In recent years, an increasing number of studies have reported that lncRNAs are involved in regulating the expression of OCT4, the key “stemness” transcription factor, in various ways; moreover, many lncRNAs have been recognized as downstream molecules of OCT4, which are reviewed in this paper.

## LncRNAs regulate OCT4 expression at the transcriptional level

### OCT4 pseudogenes

A pseudogene is a copy of a gene that has lost its ability to code for protein. LncRNAs produced by pseudogenes have similar base sequences to parental gene transcripts and can compete with parent gene transcripts for microRNA and RNA-binding proteins or can be cleaved into endogenous small molecule interfering RNA (siRNA) to regulate the expression of parental genes [15].

The OCT4-encoding gene *POU5F1* has 8 pseudogenes (*hPOU5F1P1-8*) in Homo sapiens and 5 pseudogenes (*mPOU5F1P1-5*) in Mus musculus [16]. Scarola et al. [16, 17] reported that OCT4-pg4 (transcript of *mPou5F1P4*) can change its spatial conformation by recruiting the RNA-binding protein FUS, exposing specific functional domains and binding to histone methyltransferase SUV39H1 to form the mOCT4P4-FUS-SUV39H1 complex, which then targeted the OCT4 promoter region, promoted H3K9me3 and inhibited OCT4 expression. Parental OCT4 does not have the ability to bind FUS and SUV39H1 to further regulate its transcription. Human OCT4-pg3 (a transcript of *hPOU5F1P3*) has the same function, indicating the evolutionarily conserved nature of the OCT4 pseudogenes. Hawkins et al. [18] reported that mouse OCT4-pg5 (transcript of *mPou5F1P5*) binds to the antisense strand of *mPOU5F1* complementarily, guides histone methylation transferase Ezh2 to the promoter region, elevates local H3K9me2 and H3K27me3 levels, and silences OCT4 expression. In addition, some lncRNAs indirectly regulate OCT4 expression by promoting the expression of pseudogenes. For example, lncRNA colon cancer associated transcript 2 (CCAT2), which is located on 8q24.21, was reported to upregulate OCT4 expression by transactivated hPOU5F1P1 in human triple negative breast cancer [19].

### Oplr16, Osblr8 and Peblr20

Although Yamanaka et al. [4] won the Nobel Prize for reprogramming adult cells to iPSCs through the OKSM (OCT4/SOX2/KLF4/C-MYC) scheme; in fact, less than 1% of the transfected cells were reprogrammed to iPSCs in the experiment. Further studies showed that the endogenous expression of OCT4 was a key factor affecting the reprogramming efficiency [20]. Later, researchers reported some mechanisms affecting the endogenous expression of OCT4. For example, Zhang et al. [21] reported that structural maintenance of chromosomes 1 (SMC1) mediated the folding of the chromosome region where OCT4 is located, forming the inner ring to aggregate the OCT4 promoter and enhancers distal from the promoter, facilitating the formation of transcription complexes. Pastor et al. [22] reported that ten-eleven translocation (TET) proteins upregulated the endogenous expression of OCT4 by reducing the methylation level of the OCT4 promoter. Li's team recently reported three lncRNAs promoting OCT4 expression through the abovementioned mechanism: OCT4 promoter-interacting long noncoding RNA 16 (Oplr16) [23], OCT4-SOX2 binding long noncoding RNA 8 (Osblr8) [24] and Pou5F1 enhancer binding lncRNA 20 (Peblr20) [25].

Oplr16 is a 629 bp lncRNA that is located on chromosome 17 in mice and chromosome 6 in rats. Li's team [23] reported that Oplr16 was highly expressed in iPSCs and ESCs but not in mature tissues or cell types, including arteries, brain, cerebellum, fat, kidney, liver, lung, spleen, heart and fibroblasts. Overexpression of Oplr16 can improve the efficiency of fibroblast reprogramming into iPSCs. In situ reverse transcription of chromatin RNA sequencing revealed that Oplr16 specifically binds the Oct4 promoter. After binding with Oplr16, OCT4 expression is promoted through two mechanisms: recruiting SMC1 to form an intrachromosomal loop and recruiting the DNA demethylase TET2 to reduce the methylation levels of the OCT4 promoter region. Osblr8 [24] is 210 bp long and located on human chromosome 3. Binding of Osblr8 at the OCT4 promoter region causes the 5'-terminal enhancer and 3' -terminal enhancer to simultaneously fold to the promoter region, forming an intrachromosomal loop. Furthermore, Osblr8 not only recruits TET family members but also regulates their expression. Peblr20 [25] also promotes OCT4 expression through the above mechanisms.

## LncRNAs regulate OCT4 expression at the posttranscriptional level

### DANCR

Differentiation antagonizing non-protein coding RNA (DANCR) is located at human 4q12. Recently, abnormal elevation of DANCR has been reported to be associated with a variety of human malignancies, such as breast cancer [26], ovarian cancer [27], colon cancer [28] and osteosarcoma [29]. Tao et al. [26] reported that DANCR overexpression promotes the proliferation, invasion and metastasis of the human breast cancer cell lines MCF-7 and MDA-MB-231. Silencing DANCR by siRNA significantly downregulated the expression of OCT4. miR-216a-5p not only binds and degrades DANCR but also targets OCT4 mRNA. Therefore, DANCR competitively binds to miR-216a-5p to upregulate OCT4 expression, thereby affecting the biological behaviours of breast cancer. In addition, DANCR has also been reported to competitively bind to other miRNAs targeting OCT4 mRNA, such as miR-145 [27], miR-125 [28] and miR-335 [29], suggesting that DANCR is extensively involved in the regulation of OCT4 expression at the posttranscriptional level.

### SLCO4A1-AS1

Solute carrier organic anion transporter family member 4A1-antisense RNA 1 (SLCO4A1-AS1) is located on the long arm of human chromosome 20 (20q13.33) and complementarily binds to miR-335-5p, which downregulates *OCT*4 expression at the posttranscriptional level [12, 29]. Yang et al. [12] reported that SLCO4A1-AS1 is upregulated in bladder cancer. Silencing lncRNAs can inhibit the proliferation, colony formation, invasion and metastasis of bladder cancer cell lines, while overexpression of OCT4 can remove the effect of silencing SLCO4A1-AS1.

### Linc00337 and LincRNA-ROR

miR-145 targets and degrades OCT4 mRNA, while both long intergenic non-protein coding RNA 337 (Linc00337) and long intergenic non-protein coding RNA-regulator of reprogramming (LincRNA-ROR) bind to miR-145 [27, 30–32]. Linc00337 is located on the short arm of human chromosome 1 (1p36.31). Han et al. [31] reported that the expression of Linc00337, OCT4 and other stemness markers, such as SOX2 and NANOG, in CD44^+^/CD24^low/−^ sphere-forming cells (SFCs) isolated from HeLa cell lines was much higher than that in HeLa cells. After Linc00337 was knocked down in CD44^+^/CD24^low/−^ SFCs by the shRNA technique, OCT4 mRNA was degraded by miR-145, which weakened the stem cell characteristics of SFCs, decreased cell viability, decreased sphere-forming ability, arrested the cell cycle in G0/G1 phase, and increased the sensitivity to chemotherapeutic drugs, including cisplatin, adriamycin and epirubicin. LincRNA-ROR, gene of which is located on the long arm of human chromosome 1 (8q21.31), was reported to upregulate OCT4 expression by competitively binding to miR-145 and play a biological role in human prostate cancer stem cells and human colon cancer cells by Liu et al. [30] and Yan et al. [32].

### SNHGs

Small nucleolar RNA host genes (SNHGs) are named for the small molecule nucleolar RNA they encode. Among the 232 members of this family, 15 members, including SNHG1/2/3/7/14/20, belong to lncRNAs and have been reported to be associated with a variety of human malignant tumours [33]. SNHG3 is located on human chromosome 1p35.3. In cisplatin-resistant gastric cancer cells, interleukin-6 promoted the expression of SNHG3, which was positively correlated with drug resistance and “stemness” (partially manifested by overexpression of OCT4 and other stem cell markers), and the expression of OCT4 was significantly downregulated when SNHG3 was knocked out [34]. SNHG3 may indirectly promote the expression of OCT4 by competitively binding to miR-3619-5p to relieve the latter's inhibition of the expression of ARL2, a member of the Ras protein family. In mesenchymal stem cells, SNHG3 inhibition also downregulated OCT4 expression and promoted cell differentiation [35]. SNHG7, the gene located on human chromosome 9q34.3, promotes breast cancer by competitively binding miR-34a and activating Notch-1 signalling [36]. Li et al. [37] reported that knock down of SNHG7 through the shRNA technique inhibited OCT4 expression as well as sphere formation in vitro. This effect was relieved by miR-34a supplementation. The SNHG20 gene is located on human chromosome 17q25.2. SNHG20 competitively binds to miR-197, which is also targeted by Lin28. In oral squamous cell carcinoma cell lines, si-SNHG20 downregulated the expression of *LIN28* and OCT4, while the miR-197 inhibitor upregulated the expression of OCT4. Cotransfection of si-SNHG20 and miR-197 had no significant effect on the expression of OCT4 [38].

## LncRNAs regulated by OCT4

### LX8-SINE B2

Recently, Chen et al. [39] discovered LX8-SINE B2, a lncRNA with a promising role as a stemness marker. *LX8-SINE B2*, with a length of 734 bp, is encoded by three exons and is named because its first and third exons overlap with the gene regions of LX8 and SINE B2, respectively. The expression of *LX8-SINE B2* decreases gradually during the differentiation of ESCs and it is not expressed in mouse embryonic fibroblasts. The Transcription Factor Binding Site (TFBS) of OCT4 and SOX2 exists on the promoter of *LX8-SINE B2*. Knocking down OCT4 or *SOX2* significantly inhibits promoter activity and downregulates the expression of this lncRNA.

### NEAT1

Jen et al. [13] reported that OCT4 could bind to the promotor of nuclear paraspeckle assembly transcript 1 (NEAT1) and the enhancer of metastasis associated lung adenocarcinoma transcript 1 (MALAT1). In lung cancer cells, overexpression of OCT4 upregulated the transcriptional activity of the NEAT1 promoter and MALAT1 enhancer, as well as their expression. Silencing OCT4 by siRNA downregulated the expression of *NEAT1* and *MALAT1*. Silencing *NEAT1* and *MALAT1* also inhibited tumour proliferation, invasion and metastasis induced by overexpression of OCT4. Jen et al. [13] also discovered that co-overexpression of OCT4, *NEAT1* and *MALAT1* was an independent factor of poor prognosis by univariate Cox regression analysis of 124 lung cancer patients. In addition, the expression of *NEAT1.1*, an isomer of *NEAT1*, was significantly increased in bladder cancer cells, and OCT4 bound to and upregulated the expression of *NEAT1.1* in bladder cancer cells sensitive to cisplatin treatment [40].

## LncRNAs mutually regulated with OCT4

### ES1

ES1 (LINC01108), located on human chromosome 6p23, is involved in maintaining the pluripotency of ESCs. OCT4 binds its TFBS distributed near the ES1 transcription start site (TSS) and promotes ES1 transcription [41]. Mohamed et al. [42] reported that the expression level of ES1 in human breast cancer tissues was significantly higher than that in adjacent normal tissues, and its expression was positively correlated with breast cancer grade and stage. In breast cancer cell lines, knock down of ES1 inhibited cell proliferation and epithelial mesenchymal transformation and arrested the cell cycle but also inhibited apoptosis and senescence. Interestingly, knock down of ES1 downregulated the expression of OCT4 and microRNAs downstream of OCT4 (miR-302 and miR-106b), suggesting that there may be a feedback regulatory loop between ES1 and OCT4.

### MIAT

Myocardial infarction-associated transcript (MIAT), located on human chromosome 22q12.1, is named because its transcription levels are associated with the risk of cardiac infarction [43]. The TFBSs of OCT4 are widely distributed near the TSS of MIAT. Knock down of OCT4 by RNAi technology significantly inhibited the transcription of MIAT, while knock down of MIAT significantly downregulated the expression of OCT4, suggesting that there may be a positive feedback regulatory loop between OCT4 and MIAT [44]. This feedback regulatory loop was reported again in invasive chronic lymphoblastic leukaemia [45] and breast cancer [46]. Recently, Yao et al. [47] reported that OCT4-induced MIAT transcriptional activation promoted 5-FU chemotherapy resistance in colon cancer.

### MALAT1

As mentioned above, Jen et al. [13] found that OCT4 promotes MALAT1 expression by binding MALAT1 enhancers. OCT4 mRNA is a target of miR-20b-5p, and MALAT1 competitively binds to miR-20b-5p, thereby relieving the suppression of OCT4 expression by the latter. Moreover, si-MALAT1 was reported to reduce OCT4 expression in lung adenocarcinoma and suppress tumour growth, metabolism and stemness [48].

## Cis-regulatory elements shared with OCT4

### PSORS1C3

In addition to the transcript itself, genomic cis-regulatory elements of lncRNAs, such as promoters or enhancers, can also exert regulatory effects on adjacent genes [49]. For example, psoriasis susceptibility 1 candidate 3 (PSORS1C3) is mostly reported in psoriasis-related studies, hence its name [50]. *PSORS1C3* and OCT4 are located in the adjacent regions of human chromosome 6. Azad et al. [51, 52] reported that the promoter of *PSORS1C3* can act as an enhancer of OCT4, and its deletion leads to a significant decrease in the expression of OCT4. However, when binding to the glucocorticoid receptor, it inhibits OCT4 transcription. In renal cell carcinoma, hypoxia-inducible factor simultaneously promotes the transcription of *PSORS1C3* and OCT4 by binding to the above promoter [53].

## Conclusion

**Fig. 1:**
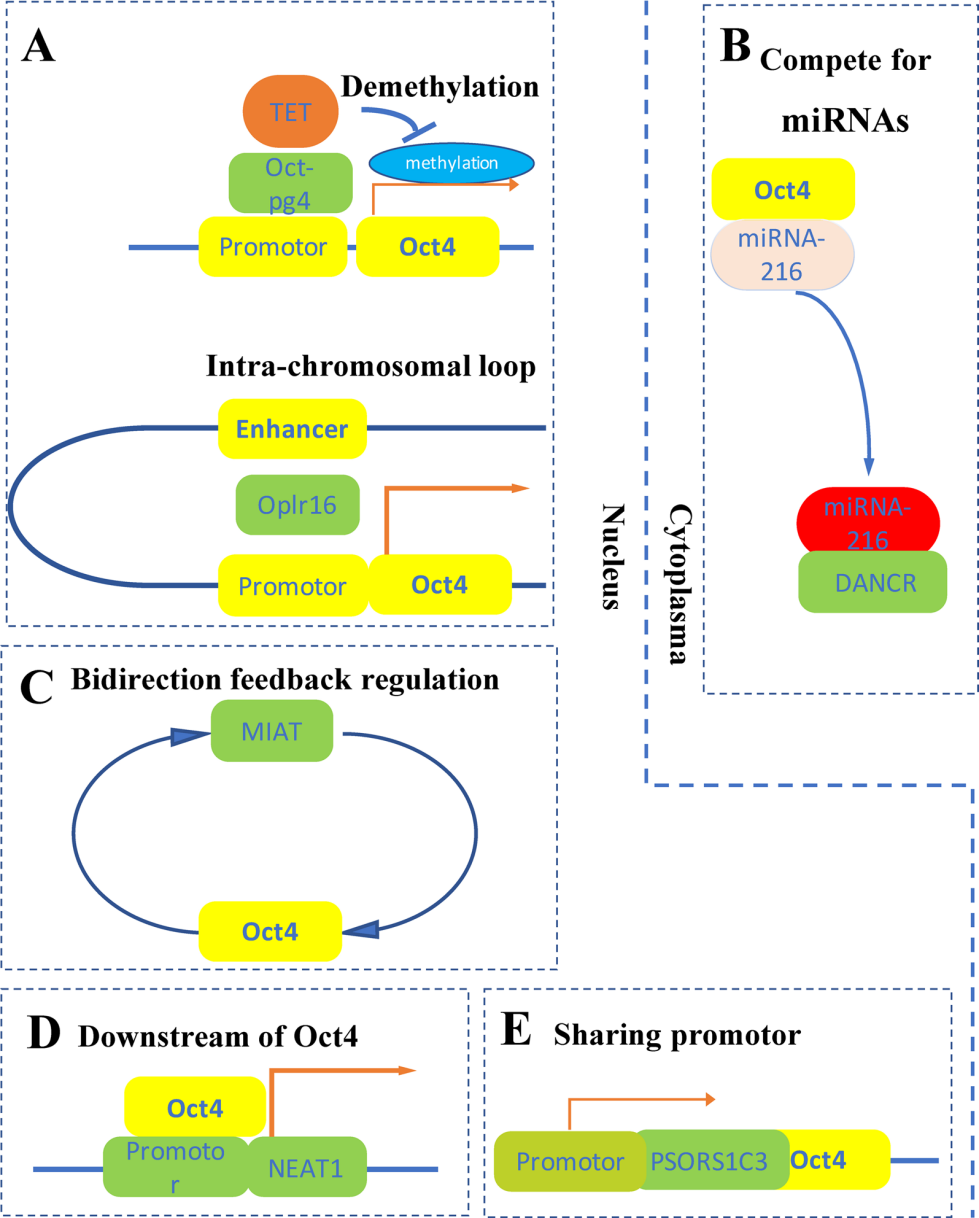
LncRNAs participate in the regulation of OCT4 expression through multiple mechanisms. A. LncRNAs including Oct4-pg4 binding to the OCT4 gene promoter change the degree of local methylation. Oplr16 mediates the formation of the intrachromosomal loop and gathers OCT4 cis-acting elements scattered in different parts of chromosomes. B. LncRNAs including DANCR inhibit OCT4 downregulation at the posttranscriptional level by competing with OCT4 mRNA to bind miRNAs. C. LncRNAs including MIAT and OCT4 regulate each other's expression through a feedback loop. D. LncRNAs including NEAT1 are a downstream molecule of OCT4. E. LncRNAs including *PSORS1C3* share a promoter with OCT4

In this paper, we reviewed the various ways in which lncRNAs regulate OCT4 expression (Fig. 1, Table 1). Oct4-pg4/5, Oplr16, Osblr8 and Peblr20 binding to the OCT4 promoter change the degree of local methylation. Oplr16, Osblr8 and Peblr20 also mediate the formation of the intrachromosomal loop and gather the OCT4 cis-acting elements scattered in different parts of chromosomes. DANCR, SLCO4A1-AS1, LincRNA-ROR, Linc00337 and SNHG3/7/20 inhibit OCT4 downregulation at the posttranscriptional level by competing with OCT4 mRNA to bind miRNAs. *PSORS1C3* shares a promoter with OCT4*.* ES1, MIAT, MALAT1 and OCT4 regulate each other's expression through a feedback loop. In addition, LX8-sine B2 and NEAT1 are downstream targets of OCT4.

**Table 1:** Summary of OCT4 associated lncRNAs

LncRNA	Location (Human)	Mechanism	Cell line	Nude mice xenograft tumour model	Human sample	Refs.
mmu-OCT4-pg4	Mice	RNA-binding protein FUS; Histone methyltransferase SUV39H1	mice ESC cell(Isolated from C57BL/6 mouse embryos)	–	–	[13, 14]
hsa-OCT4-pg3	12p13.31	Same as above	OVACR-3	–	–	[14]
mmu-OCT4-pg5	Mice	Histone methyltransferase Ezh2	Breast cancer cell line: MCF-7	–	–	[15]
CCAT2	8q24.21	OCT4-pg1; miR-205	Breast cancer cell line: MCF-7, MDA-MB-231, etc.	MDA-MB-231	Breast cancer	[16]
Oplr16	Mice	Intrachromosomal loop; DNA demethylase TETs	iPSC (reprogramed from mice fibroblast by OSKM)	–	–	[20]
Osblr8	Mice	Same as above	iPSC (same as above), mice ESC cell line E14	–	–	[21]
Peblr20	Mice	Same as above	iPSC(same as above)	–	–	[22]
DANCR	4q12	miR-216a-5p; miR-335; miR-145	Breast cancer cell line: MCF-7, MDA-MB-231, etc.; urothelium cell line SV-HUC-1	MDA-MB-231	Breast cancer	[5–7, 23]
SLCO4A1-AS1	20q13.33	miR-335-5p	Bladder cancer cell lines: EJ, T24 andRT4;	T24	Bladder cancer	[8]
Linc00337	1p36.31	miR-145	Cervical cancer cell lines: HeLa, SiHa, CaSki, etc.; sphere-forming subpopulation isolated from HeLa cells	sphere-forming subpopulation	Cervical cancer	[9]
LincRNA-ROR	18q21.31	miR-145	CD44^+^/CD133^+^ subpopulation isolated from Prostate cancer cell lines Du145 and 22RV1	CD44^+^/CD133^+^ subpopulation	Prostate cancer	[24, 25]
SNHG3	1p35.3	miR-3619-5p	Gastric cancer cell lines SGC7901 and BGC823; mice ESC cell line AB2.2	–	Gastric cancer	[27, 28]
SNHG7	9q34.3	miR-34a	Breast cancer cell line: MCF-7, MDA-MB-231, etc.	–	Breast cancer	[29, 30]
SNHG20	17q25.2	miR-197, Lin28	Oral squamous cell carcinoma cell lines SCC9, SCC15, SCC25, etc.	SCC15	Oral squamous cell carcinoma	[31]
LX8-SINE B2	Mice	Regulated by OCT4	mice ESC cell line E14	–	–	[32]
NEAT1	11q13.1	Regulated by OCT4; miR-204-5p	Lung cancer cell lines A549 and CL1-0; Bladder cancer cell line T24	Ovariectomized obese mouse model induced by high fat diet	Lung cancer	[10, 33]
ES1	6p23	mutually regulated with OCT4	人Breast cancer cell line: MDA-MB-231, SKBR3, etc.	–	Breast cancer	[34, 35]
MIAT	22q12.1	Same as above	mice ESC cell line E14; Leukaemia/Lymphoma cell lines BALL, DLBL, etc. Breast cancer cell line: MCF-7, MDA-MB-231, etc.	–	Leukaemia; Breast cancer	[37–39]
MALAT1	11q13.1	Same as above	Lung cancer cell lines A549 and CL1-0; Colorectal cancer cell lines COLO205, HCT‐116, etc.	HCT‐116	Lung cancer	[10, 41]
PSORS1C3	6p21.33	Share promotor with OCT4	Teratoma cell line NT2; A549; Kidney cancer cell lines CRL-1932/1611	–	Clear cell carcinoma of kidney	[44–46]

The abovementioned lncRNAs that regulate or are regulated by OCT4 expression not only have important research and application value in the fields of iPSCs, ESCs and CSCs but also have the potential to become diagnostic markers, prognostic predictors and therapeutic targets of various human malignant tumours. For example, overexpression of Oplr16 can be used to improve reprogramming efficiency in the process of reprogramming adult cells to iPSCs through the OKSM cocktail [23]. The coexpression of OCT4, *NEAT1* and *MALAT1* can also be used to predict the prognosis of lung cancer patients [13]. Detection of MALAT1 in peripheral blood or urine samples can be used for the early diagnosis of lung cancer [54] and prostate cancer [55], respectively. A large number of studies have reported that targeted inhibition of DANCR, SLCO4A1-AS1, Linc00337, SNHG7/20, NEAT1, MALAT1, ES1 or MALAT1 can reduce the degree of malignancy of tumours at the cellular level and even in animal models. Therefore, targeting the above lncRNAs by siRNA, shRNA or antisense oligonucleotides has the potential to treat human malignancies.

## Data Availability

Not applicable.

## Abbreviations

SCs: Stem cells
HSCs: Haematopoietic stem cells
ESCs: Embryonic stem cells
OCT4: Octamer-binding transcription factor 4
POU5F1: POU class 5 homeobox 1
TF: Transcription factor
iPSCs: Induced pluripotent stem cells
CSCs: Cancer stem cells
LncRNAs: Long noncoding RNAs
CCAT2: Colon cancer associated transcript 2
SMC1: Structural maintenance of chromosomes 1
TET: Ten-eleven translocation
Oplr16: OCT4 promoter-interacting long noncoding RNA 16
Osblr8: OCT4-SOX2 binding long noncoding RNA 8
Peblr20: Pou5F1 enhancer binding lncRNA 20
DANCR: Differentiation antagonizing non-protein coding RNA
SLCO4A1-AS1: Solute carrier organic anion transporter family member 4A1-antisense RNA 1
LincRNA-ROR: Long intergenic non-Protein coding RNA-regulator of reprogramming
Linc00337: Long intergenic non-protein coding RNA 337
SFCs: Sphere-forming cells
SNHGs: Small nucleolar RNA host genes
TFBS: Transcription factor binding site
NEAT1: Nuclear paraspeckle assembly transcript 1
MALAT1: Metastasis associated lung adenocarcinoma transcript 1
TSS: Transcription start site
MIAT: Myocardial infarction-associated transcript
PSORS1C3: Psoriasis susceptibility 1 candidate 3
